# CYP1A1/20-HETE/GPR75 Axis-Mediated Arachidonic Acid Metabolism Dysregulation in H-Type Hypertension Pathogenesis

**DOI:** 10.3390/ijms26135947

**Published:** 2025-06-20

**Authors:** Hangyu Lv, Lingyun Liu, Baoling Bai, Kexin Zhang, Qin Zhang

**Affiliations:** Beijing Municipal Key Laboratory of Child Development and Nutriomics, Capital Children’s Medical Center, Capital Medical University, Capital Institute of Pediatrics, Beijing 100020, China; lhy2990599177@163.com (H.L.); 15963782562@163.com (L.L.); baoxiang8802@126.com (B.B.); kexinzhang02@163.com (K.Z.)

**Keywords:** H-type hypertension, homocysteine, arachidonic acid, 20-HETE, CYP1A1, Gpr75

## Abstract

This study aims to explore the pathogenic mechanism of H-type hypertension. A rat model of H-type hypertension was established through high-methionine dietary intervention, with subsequent folic acid administration. Untargeted serum metabolomic profiling identified a significant reduction in arachidonic acid (AA) levels in the methionine-enriched group, which were effectively normalized following folic acid supplementation. Transcriptomic analysis revealed methionine-induced upregulation of AA pathway-associated genes *Cyp1a1* and *Gpr75*. In contrast, after the intervention with folic acid, a downregulation of these genes was observed. These findings were corroborated through Western blotting and RT-qPCR validation. In vitro studies using EA.hy926 endothelial cells demonstrated that methionine exposure significantly elevated CYP1A1 expression. Furthermore, methionine stimulation induced marked upregulation of GPR75 and its downstream signaling components (NRAS, MEK1, and ERK1). Population-level evidence from the U.S. NHANES database substantiated significant correlations between essential fatty acids (AA, LA, and GLA) and H-type hypertension prevalence. Our research findings suggest that the CYP1A1/20-HETE/GPR75 axis-mediated dysregulation of AA metabolism may be one of the key pathological mechanisms of H-type hypertension. The research results provide clues for the discovery of new therapeutic targets for H-type hypertension.

## 1. Introduction

H-type hypertension is characterized by essential hypertension in conjunction with hyperhomocysteinemia (HHcy), specifically defined as essential hypertension with elevated plasma homocysteine (Hcy) levels of ≥10 μmol/L [1]. In China, it is estimated that approximately 75% to 80.3% of adult individuals with hypertension are diagnosed with H-type hypertension [2]. This condition is associated with an increased risk of target organ damage, affecting the heart, brain, and kidneys [3]. Moreover, the incidence of cardiovascular diseases in patients with H-type hypertension is approximately five times greater than in those with simple hypertension [4]. A significant positive correlation exists between Hcy concentration levels and the incidence of hypertension [5,6]. Specifically, for every 5 μmol/L increase in total plasma Hcy levels, the risk of cardiovascular disease rises by approximately 9% [7,8]. Furthermore, when total plasma Hcy concentrations exceed 18 μmol/L, the incidence of hypertension increases threefold [9]. However, the mechanism by which HHcy leads to hypertension is not yet fully understood. Research on the mechanism of HHcy-induced hypertension will help us gain a deeper understanding of hypertension, providing a theoretical basis for the early diagnosis of hypertension and the identification of new intervention targets.

Recent studies have highlighted the contribution of lipid mediators to the pathogenesis of hypertension. Arachidonic acid (AA), a polyunsaturated fatty acid released from membrane phospholipids, is metabolized by cytochrome P450 (CYP) enzymes into several bioactive compounds. Among them, 20-hydroxyeicosatetraenoic acid (20-HETE), primarily generated by the CYP4A and CYP4F families, has been shown to modulate vascular tone, renal sodium handling, and inflammatory responses [10,11]. In addition, a study has shown that Cyp1a1 may metabolize AA into 20-HETE [12]. But there have been no reports of *CYP1A1* gene involvement in H-type hypertension.

Multiple studies have investigated the role of Hcy in elevated blood pressure and end-organ damage by constructing rat models, such as a high methionine diet, gene knockout, and alternative methods [13,14]. The diet plays a major role in the pathogenesis of hypertension, with particular emphasis on diets rich in methionine. Therefore, in animal models of H-type hypertension, high methionine diet feeding is one of the most commonly used methods [13,15].

Folic acid is a necessary cofactor in Hcy metabolism. Several studies have shown that folic acid lowers blood pressure (BP) and Hcy concentrations in hypertensive rats. Many clinical trials have suggested that folic acid also has beneficial effects on BP by increasing nitric oxide synthesis in endothelial cells or by reducing plasma Hcy concentrations. Two prospective cohort studies showed that a higher total folate intake was associated with a decreased risk of incident hypertension, particularly in younger women. However, the mechanism of folic acid treatment for hypertension is not yet fully understood.

Therefore, this study uses animal models of H-type hypertension induced by high methionine and folic acid treatment to explore the pathogenic mechanism of H-type hypertension and the possible mechanism of folic acid treatment for H-type hypertension, providing clues for discovering new targets for the treatment of hypertension.

## 2. Results

### 2.1. Successful Construction of the H-Type Hypertension and FA Treatment for Hypertension Model

**Figure 1:**
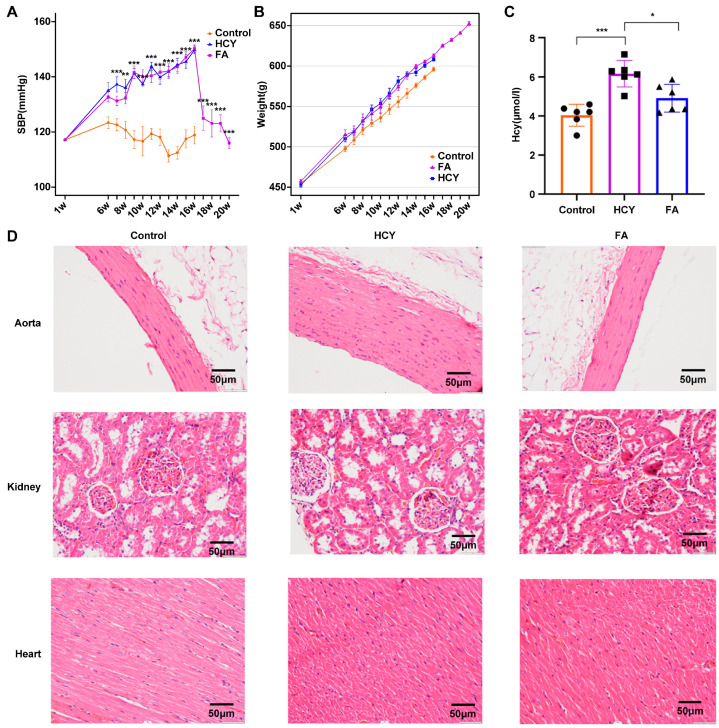
The successful construction of HHcy hypertension rat model. (**A**) The changes in systolic (SBP) of SD rats in a normal diet (control, *n* = 6), a high methionine diet (HCY, *n* = 6), and a diet high in methionine plus a folic acid diet (FA, *n* = 6) group measured by tail cuff. w: week. (**B**) The body weight of the three groups of rats throughout the intervention period. (**C**) The changes in HCY levels of SD rats in a normal diet (control, *n* = 6), a high methionine diet (HCY, *n* = 6), and a diet high in methionine plus a folic acid diet (FA, *n* = 6). Circles represent the rats in the Control group; squares represent the rats in the Hcy group; and triangles represent the rats in the FA group. (**D**) Immunohistochemical results of the aorta, kidney, and heart. The values were expressed as the mean ± SE. * indicates *p* < 0.05, ** indicates *p* < 0.01, and *** indicates *p* < 0.001.

To investigate the pathogenesis of H-type hypertension, we constructed a model of H-type hypertension induced by high methionine and a model of H-type hypertension treated with folic acid (which can reduce Hcy levels). Eighteen rats were randomly divided into the three groups described above. The weekly changes in blood pressure in the three groups are shown in Supplementary Figure S1. We found that SBP in the HCY group gradually increased upon feeding and reached a mean value of 149.83 ± 3.13 mm Hg in the 16th week, which was significantly higher than that in the control group (SBP: 120 ± 5.97 mm Hg). Starting from the 16th week, folate was fed to treat hypertension. After folate was added, the blood pressure decreased, reaching a minimum value by the 20th week (SBP: 116.16 ± 4.36 mm Hg) (Figure 1A). These results suggest that a high methionine diet can increase blood pressure in rats, and dietary supplementation of folic acid can treat hypertension in rats. It is worth noting that throughout the entire experimental period, the high methionine diet group had slightly higher body weight than the normal group, but the difference was not statistically significant (Figure 1B). Hcy concentrations were significantly different between the control and HCY groups, and there was considerable variation in Hcy concentrations between the HCY and FA groups (Figure 1C).

Furthermore, the effect of a high methionine diet on the aorta, kidney, and heart in rats was evaluated by hematoxylin–eosin staining. In the control group, the aortic thickness was normal, the cardiomyocytes were neatly arranged and tightly connected, the renal tissue was uniformly stained, and the renal tubular structure was normal and clear (Figure 1D). In the HCY group, the aortic thickness was increased, many inflammatory cells were observed in the glomerular capillary network, and in some fibrocytes, the renal interstitium was mildly fibrotic, and the cardiomyocytes were disorganized with loose intercellular connections. The histopathological injury of rats that were treated with folic acid was reduced. These results indicated that a high methionine diet led to elevated Hcy concentrations, elevated blood pressure, and vascular injury, whereas a folic acid diet ameliorated these adverse consequences.

### 2.2. Plasma Metabolomics Analysis Between Three Groups of Rats

**Figure 2:**
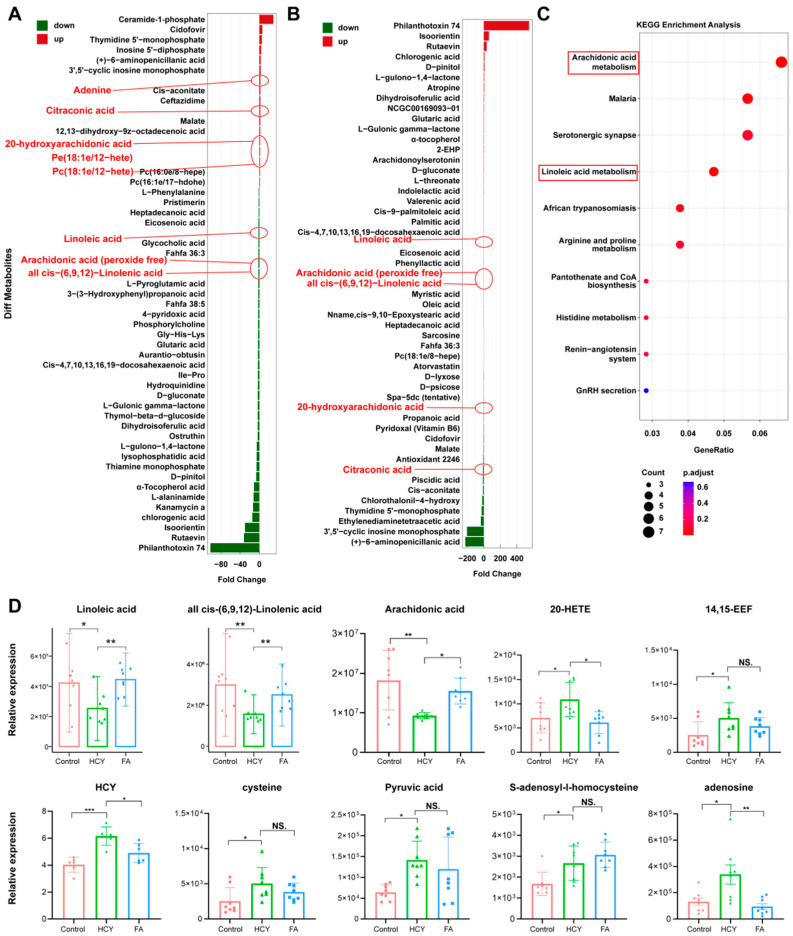
Comparison of differential metabolites among the control group, HHcy hypertension group, and FA treatment group in rats. (**A**,**B**) Bar plots displaying the fold change of differential blood metabolites in the HCY (**A**) and FA (**B**) groups compared with the control group. Key altered metabolites such as linoleic acid, arachidonic acid (peroxide free), all cis-(6,9,12)-linolenic acid, citraconic acid, and 20-hydroxyarachidonic acid are highlighted. (**C**) KEGG enrichment analysis showing that arachidonic acid metabolism and linoleic acid metabolism are significantly enriched pathways. Dot size indicates the number of involved metabolites, and color reflects the adjusted *p*-value. (**D**) Relative expression of key metabolites in plasma, including fatty acids (linoleic acid, γ-linolenic acid, arachidonic acid), 20-HETE, and HCY-related compounds. Data are presented as mean ± SD; * *p* < 0.05, ** *p* < 0.01, and *** *p* < 0.001. NS: not significant.

To discover differential metabolites and provide clues for studying H-type hypertension, we conducted a non-targeted metabolomics study on the serum of three groups of rats. Figure 2A shows the metabolites with higher ranking differences between the normal group and the Hcy group. Figure 2B shows the metabolites with higher ranking differences between the Hcy group and FA group. Among them, the level of adenine, citraconic acid, S-adenosyI-I-homocysteine (SAH), cysteine, pyruvic acid, and arachidonic acid (AA) related metabolites were significantly changed (Figure 2A,B). AA and its precursor γ-linoleic acid [cis-(6,9,12)-linoleic acid] were decreased in the HCY group. AA metabolites, 20-hydroxyeicosatetraenoic acid (20-HETE), and 14,15-epoxyeicosatrienoic acid (14,15-EET) were increased in the HCY group. After folate treatment, these metabolites showed the opposite trend. Furthermore, we found that the AA pathway was significantly enriched according to KEGG pathway analysis (Figure 2C). Here, we showed some of the important differential metabolites among these three groups in Figure 2D. The top five plots show the levels of AA, linoleic acid, γ-linolenic acid, 20-HETE, and 14,15-EET. The bottom five plots show the levels of HCY, cysteine, pyruvate, adenosine, and SAH. Heatmaps comparing differential metabolites between the control group and the high methionine group are presented in the Supplementary Materials. The significant changes observed in AA and its metabolites led to an investigation of the genes causing differential metabolite changes (Supplementary Figure S1).

### 2.3. Cyp1a1/Gpr75 in Aorta May Mediates Abnormal Arachidonic Acid Metabolism

**Figure 3:**
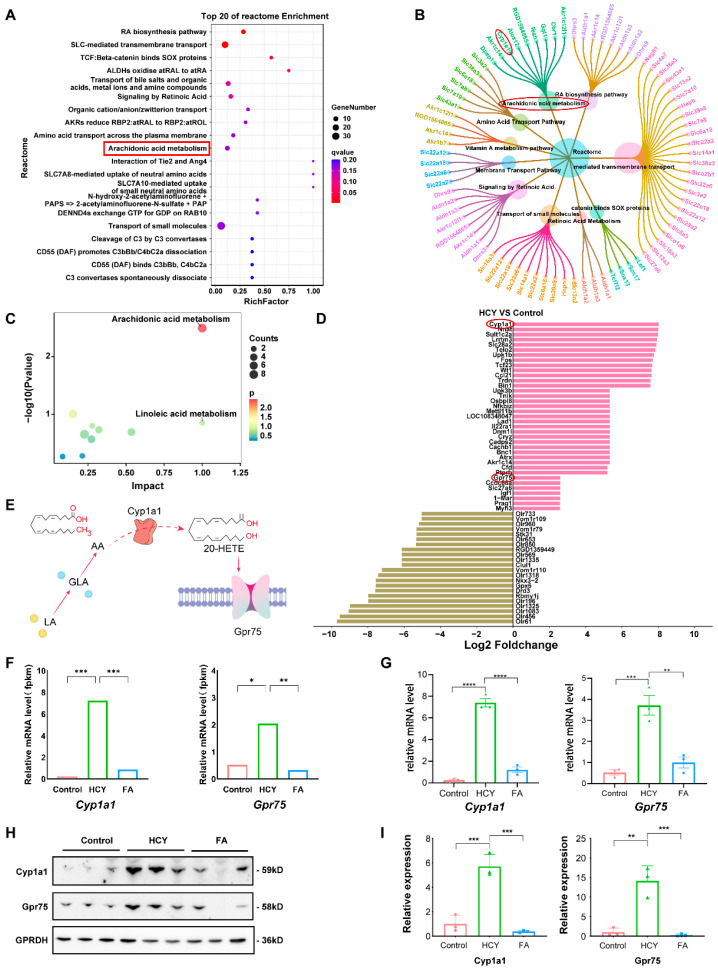
*Cyp1a1*/*Gpr75* in the aorta may mediate abnormal arachidonic acid metabolism. (**A**) Reactome pathway enrichment analysis of DEGs with the arachidonic acid metabolism pathway significantly enriched (highlighted in red). (**B**) Gene-pathway network visualization showing enriched biological pathways. The arachidonic acid metabolism and *Cyp1a1* are marked. (**C**) The figure represents the enrichment pathway for transcriptome and metabolome co-analysis. Horizontal: The impact indicator of the pathway; the higher the right, the higher the “importance” of the pathway in the network. Longitudinal axis (−log10(*p* value)): Statistically significant; the higher the difference, the more significant the difference. (**D**) Generalized fold change plot of significantly altered genes in the arachidonic acid metabolism pathway between HCY and control groups. (**E**) Schematic diagram of the relationship between *Cyp1a1* and *Gpr75* in the AA pathway. (**F**) Transcriptome sequencing showing significant upregulation of *Cyp1a1* and *Gpr75* mRNA expression in the HCY group and normalization after folic acid. (**G**) Quantitative RT-PCR analysis showing significant upregulation of *Cyp1a1* and *Gpr75* mRNA expression in the HCY group and normalization after folic acid (FA) treatment. (**H**) Western blot validation of Cyp1a1 and Gpr75 protein expression levels with GAPDH used as a loading control. (**I**) Densitometric quantification of Western blot bands for *Cyp1a1* and *Gpr75*, confirming significant overexpression in the HCY group and suppression by FA. Data are presented as mean ± SD. Statistical significance: * *p* < 0.05, ** *p* < 0.01, *** *p* < 0.001, and **** *p* < 0.0001.

Previous studies have shown that endothelial cells and vascular smooth cells produce cytochrome P450 (CYP) arachidonic acid metabolites, which can affect endothelial cell function and vascular homeostasis [16]. In order to investigate the main genes causing changes in plasma AA and 20-HETE in H-type hypertension, we analyzed the transcriptome of three groups of rat aorta (control group, Hcy group, and FA group). The initial bioinformatics analysis based on the reactome pathway database suggested the involvement of the arachidonic acid pathway (Figure 3A). The petal diagram illustrates the top 10 pathways in the response group and the differentially expressed genes within these pathways. The results also focused on the arachidonic acid pathway (Figure 3B). Next, we did a combined analysis of the metabolome and transcriptome, and the results also showed significant differences in the AA pathway (Figure 3C). To further clarify which gene in the arachidonic acid pathway may play a key role, we performed a top ranking of the differentially differentiated genes in the HCY group and the control group. The results showed that *Cyp1a1* and *Gpr75* were the most significantly upregulated genes (Figure 3D). A previous study suggests that *Cyp1a1* may metabolize AA into 20-HETE [12]. Moreover, the 20-HETE signaling can affect vascular function and induce hypertension through the G-protein-coupled receptor GPR75 [17,18]. According to the previous literature and our results, we have drawn a schematic diagram to illustrate the relationship among AA, 20-HETE, *Cyp1a1*, and *Gpr75* (Figure 3E). Therefore, *Cyp1a1* and *Gpr75* might play key roles in H-type hypertension. To further validate these findings, we performed Western blot and RT-PCR assays to assess the expression levels of *Cyp1a1* and *Gpr75* (Figure 3F,G). The results showed that both mRNA and protein levels of *Cyp1a1* and *Gpr75* were significantly elevated in the HCY group compared to the control and FA groups (Figure 3H,I). Moreover, following folic acid treatment, the expression levels of these genes were markedly downregulated (both *p* < 0.05), which suggested that folate may lower blood pressure by affecting this pathway. Thus, our findings suggest that the upregulation of *Cyp1a1* and *Gpr75* may be involved in the development of H-hypertension.

### 2.4. High Level of Methionine May Be Involved in the Occurrence of Hypertension by Activating CYP1A1/GPR75/MAPK Axis

**Figure 4:**
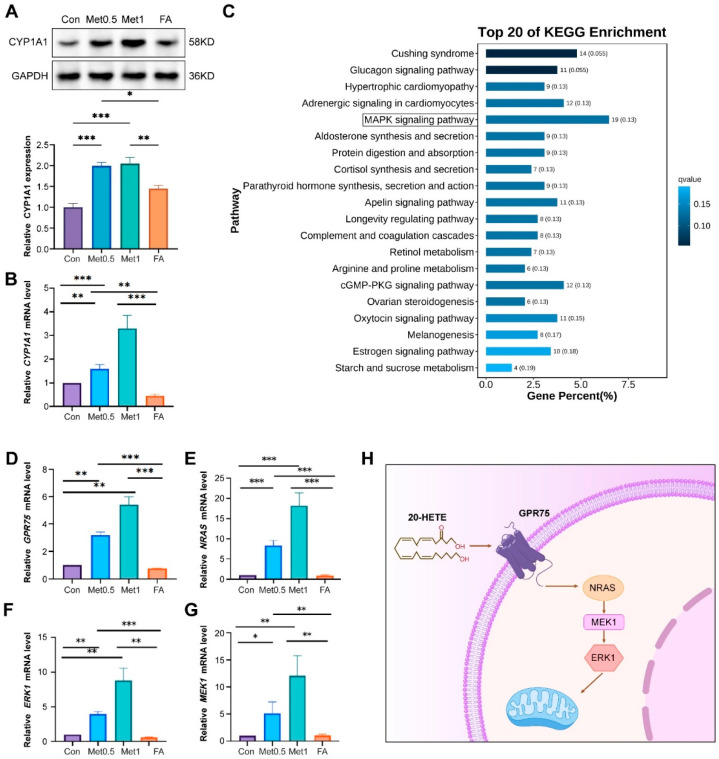
A high level of methionine activates the CYP1A1/GPR75/NRAS-MEK1-ERK1 axis in vascular endothelium. (**A**) Western blot analysis and densitometric quantification of CYP1A1 protein expression in EA.hy926 endothelial cells treated with increasing concentrations of methionine (0.5 and 1 mmol/L). Experimental groups include control (Con), 0.5% methionine diet (Met0.5), 1% methionine diet (Met1), and folic acid treatment (FA). GAPDH was used as a loading control. (**B**) Quantitative RT-PCR analysis showing mRNA expression levels of Cyp1a1 across the four groups. (**C**) KEGG enrichment analysis of differentially expressed genes between the control group and HHcy hypertension group in rats. The top 20 significantly enriched pathways are shown, with the x-axis indicating the percentage of genes involved in each pathway (gene percentage). The number of genes and q-values are displayed in parentheses. A lighter color indicates higher statistical significance. (**D**–**G**) Bar graphs showing the relative mRNA expression levels of Gpr75 (**D**), Nras (**E**), Erk1 (**F**), and Mek1 (**G**) in EA.hy926 endothelial cells under different treatments. Data are presented as mean ± SD; * *p* < 0.05, ** *p* < 0.01, and *** *p* < 0.001. (**H**) Schematic diagram illustrating the proposed mechanism by which 20-hydroxyeicosatetraenoic acid (20-HETE), a lipid metabolite, binds to GPR75 and activates the downstream MAPK cascade, including NRAS, MEK1, and ERK1.

To investigate whether the increased expression of CYP1A1 is induced by high levels of Met (which leads to elevated Hcy levels). We detected CYP1A1 protein expression levels in EA.hy926 endothelial cells under different concentrations of methionine and folate supplementation (which can reduce Hcy levels). The results showed that with the gradual increase in methionine levels, the levels of CYP1A1 also gradually increased. After folate supplementation, CYP1A1 levels returned to normal. The results suggest that Hcy levels can affect the expression level of CYP1A1 (Figure 4A,B).

Is GPR75 downstream of CYP1A1 also affected by high levels of Hcy, and through what mechanism does GPR75 participate in the occurrence of hypertension? We conducted KEGG enrichment analysis based on transcriptome data of rat aortic tissue and found that the MAPK signaling pathway is one of the most enriched pathways for differentially expressed genes (Figure 4C). Literature reports also suggest that GPR75 can participate in the occurrence of hypertension by activating the MAPK pathway. Therefore, we detected the expression changes of *GPR75* and MAPK pathway-related genes in EA.Hy926 cells after methionine and folate treatment. The results showed that in the methionine explosion group, *GPR75*, *NRAS*, *MEK1*, and *ERK1* gene levels were significantly upregulated. In the FA supplementation group, the levels of *GPR75*, *NRAS*, *MEK1*, and *ERK1* genes returned to normal (Figure 4D–G). Possible mechanisms are illustrated in Figure 4H. The above results suggest that high levels of Hcy may be involved in the development of hypertension through the CYP1A1/GPR75/MAPK axis.

### 2.5. Correlation Analysis Between AA and H-Type Hypertension Based on Population

**Figure 5:**
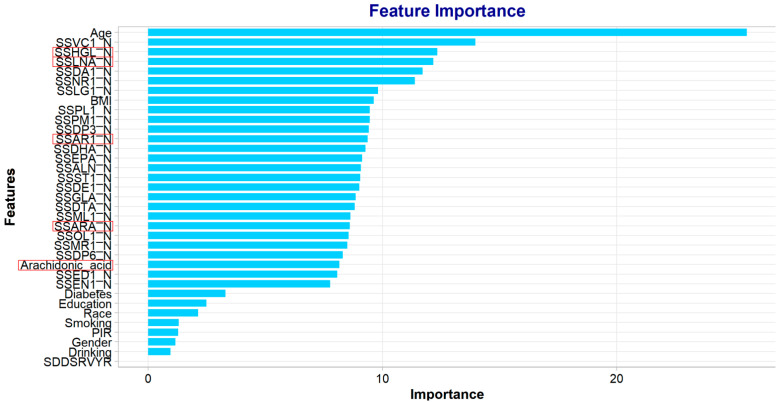
Population-based validation of AA’s pathogenic significance in H-type hypertension. The x-axis represents the importance score, while the y-axis lists the feature names. Clearly, age is the most influential predictor in the model, with a substantially higher importance score than the other features. The red boxes highlight several key metabolic features, particularly lipid-related biomarkers, including γ-linolenic acid (SSHGL-N), linoleic acid (SSLNA-N), and arachidonic acid derivatives (SSAR1-N, SSARA-N), indicating the dominant role of lipid metabolism in the model. The features highlighted in red underscore the prominent influence of lipid metabolites in the model.

The above animal experiments have shown that AA levels are associated with H-type hypertension; however, the correlation of AA in hypertensive people is still unknown. In order to further verify the correlation of AA-related metabolism in the H-hypertension population, we carried out population data mining and machine analysis based on the NHANES database, aiming to evaluate the predictive value and pathological correlation of AA and its precursors in H-hypertension from a population perspective. Building on experimental evidence implicating AA metabolic dysregulation in methionine-induced H-type hypertension, we further investigated its clinical relevance through population-level analysis. To establish translational significance, we conducted a machine learning-based feature importance analysis using a random forest classifier on the U.S. NHANES cohort (*n* = 228 meeting diagnostic criteria: hypertension with homocysteine > 10 μmol/L). This computational approach systematically evaluated the predictive contribution of serum biomarkers, particularly focusing on essential fatty acids: γ-linolenic acid (SSHGL-N), linoleic acid (SSLNA-N), and arachidonic acid derivatives (SSAR1-N, SSARA-N). Notably, the model identified AA and its metabolic precursors (linoleic acid, γ-linolenic acid) as top-ranked features associated with H-type hypertension susceptibility (Figure 5). This human population data corroborates our experimental findings, demonstrating conserved pathogenic mechanisms across species. The convergence of biochemical (AA depletion), transcriptomic *(Cyp1a1*/*Gpr75* dysregulation), and epidemiological evidence establishes AA metabolism as a critical hub in H-type hypertension pathophysiology.

## 3. Discussion

Hypertension is a global public health problem that can induce kidney and cardiovascular disease, severely affecting healthy growth and quality of life [19]. Studies have shown that H-type hypertension is an important risk factor for death in patients with cardiovascular disease [20]. In patients with H-type hypertension, antihypertensive therapy supplemented with folic acid aims to reduce Hcy concentrations [21]. However, even though most studies have suggested a close association between hypertension and Hcy concentrations, there is no clear understanding of the reasons for this relationship, and the mechanism of interaction between hypertension and Hcy concentrations is not well understood.

**Figure 6:**
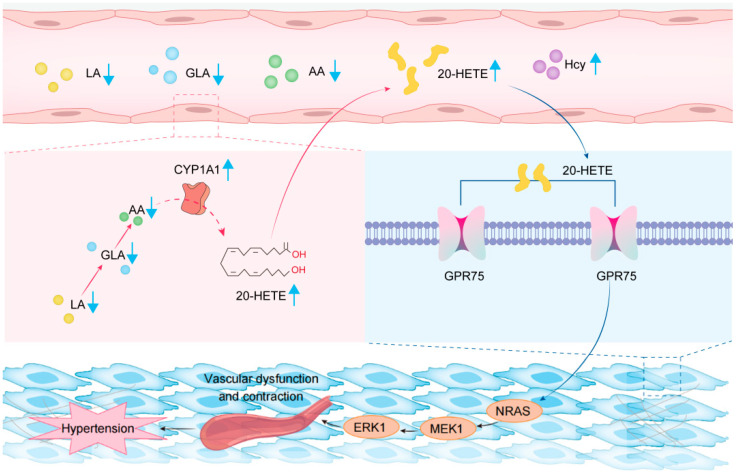
The schematic diagram of the possible mechanism of HHcy hypertension. This diagram illustrates a cascade of metabolic and vascular changes triggered by a high-methionine diet (HMD). The yellow, blue, and green spheres represent linoleic acid (LA), gamma-linolenic acid (GLA), and arachidonic acid (AA), respectively, all of which are reduced (↓) in the blood following HMD. Methionine is metabolized into homocysteine (Hcy), represented by purple circles in plasma, indicating elevated levels (↑). The central red structure represents the enzyme CYP1A1, whose activity is increased under the influence of high methionine, catalyzing the conversion of AA into 20-hydroxyeicosatetraenoic acid (20-HETE). The yellow structure represents 20-HETE, whose levels are increased (↑) in the blood as a result of this enzymatic activity. 20-HETE activates downstream signaling by binding to its receptor, GPR75, which is shown as a colorful transmembrane protein. Upon activation by 20-HETE, GPR75 initiates intracellular signaling cascades, notably the MAPK pathway. In endothelial cells, this results in elevated expression of NRAS and ERK1, with MEK1 ultimately contributing to endothelial dysfunction and the development of hypertension.

In this study, we found that the CYP1A1/20-HETE/GPR75 axis-mediated dysregulation of AA metabolism may be one of the key pathological mechanisms of H-type hypertension. Based on our data, we propose a possible mechanism, as shown in Figure 6. High levels of hcy activate CYP1A1 enzyme activity in vascular endothelial cells, leading to an increase in arachidonic acid catabolism. This causes a decrease in arachidonic acid, linoleic acid, and linolenic acid in plasma and an increase in 20-HETE. Elevated 20-HETE binds to Gpr75 receptors in vascular smooth muscle and activates the MAPK (NRAS-MEK1-ERK1) pathway, participating in the occurrence and development of hypertension.

Cytochrome P450 1A1 (CYP1A1) is a member of the cytochrome P450 superfamily of enzymes, which are primarily involved in the oxidative metabolism of xenobiotics and endogenous substrates such as fatty acids and steroids. CYP1A1 is mainly expressed in extrahepatic tissues, including the lungs, intestines, and vascular endothelium. The mechanism of CYP1A1 elevation in this study is unclear. Further experiments are needed to clarify and verify.

In our study, we observed a significant upregulation of Gpr75 expression, which was closely associated with AA metabolism. G-protein-coupled receptor 75 (GPR75) is a newly identified member of the GPCR superfamily known to play important roles in hypertension. Recent studies have revealed that 20-hydroxyeicosatetraenoic acid (20-HETE), a major metabolite of AA produced by cytochrome P450 enzymes, functions as a high-affinity endogenous ligand of GPR75 [11]. To date, three ligands for GPR75 have been identified: 20-HETE, CCL5, and RANTES [11,22].

The 20-HETE/GPR75 axis has been implicated in the pathogenesis of hypertension via activation of three key signaling pathways. The PI3K/Akt pathway is involved in vascular remodeling and endothelial dysfunction [22], the c-Src/EGFR/MAPK/NF-κB pathway increases oxidative stress and upregulates ACE expression by activating uncoupled nitric oxide synthase (NOS) [17], and the PLC/IP3/PKC pathway contributes to vascular smooth muscle contraction and blood pressure regulation [23]. In line with these findings, our study showed the upregulation of several key genes in the MAPK pathway, suggesting that GPR75 activation induced by high methionine may be involved in the development of hypertension through the MAPK pathway.

Folic acid is known to lower blood pressure indirectly by reducing homocysteine (Hcy) levels, which is supported by multiple studies [24,25]. Elevated Hcy is considered an independent risk factor for endothelial dysfunction and hypertension, and folic acid supplementation has been shown to reduce plasma Hcy levels and improve vascular function. Moreover, a study suggests that folic acid may also have a direct antihypertensive effect independent of Hcy reduction. Rapid changes in nitric oxide-mediated endothelial function were observed after supplementation with folic acid but before plasma hcy changes were detected, which implies a role for plasma hcy-independent mechanisms [26].

Our research shows that after folate supplementation, rat blood pressure decreased, Hcy levels decreased, AA metabolites returned to normal levels, and the expression level of Cyp1a1 and Gpr75 returned to normal; after simultaneous treatment with high methionine and folate in endothelial cells, gene expression on the CYP1A1/GPR75/MAPK axis returned to normal. These all suggest that folate may participate in blood pressure reduction by lowering Hcy levels and restoring CYP1A1/GPR75/MAPK axis expression levels, but the specific mechanism of folate-induced blood pressure reduction still needs to be experimentally verified.

Our research also suggests that special attention should be paid to the impact of diet on H-type hypertension. Reducing the intake of foods rich in methionine or supplementing with folic acid can effectively reduce the risk of H-type hypertension.

## 4. Materials and Methods

### 4.1. Rat Modeling

Eighteen male Sprague Dawley (SD) rats (8 weeks old) were provided by Beijing Viton Lihua Laboratory Animal Technology Co., Ltd. (Beijing, China) (Laboratory Animal Quality Certificate: No. 110011220110904328). They were housed in designated pathogen-free rooms with controlled temperature and humidity (25 °C ± 2 °C, 55% ± 5% relative humidity), a 12 h light/dark cycle, and allowed free access to water and feed. The SD rats were randomly divided into the following three groups: normal diet (control group), which received standard chow [23% protein (0.4% methionine), 12% fat, 65% carbohydrates]; high methionine diet (group HMD, standard chow + 0.77% methionine); and high methionine plus folate diet (group FA, standard chow + 0.77% methionine + 0.01% folate). The HMD group was provided methionine chow for 16 weeks. The FA group was fed the methionine chow for 16 weeks, followed by methionine FA chow for 4 weeks. All feeds were purchased from KAO Co-operative Feed Co. (Beijing, China). The rats were anesthetized with isoflurane to measure body weight and abdominal circumference, and blood samples were collected by percutaneous cardiac puncture. All rats were then euthanized by carbon dioxide asphyxiation. After death, kidney, aorta, and heart tissues were extracted, flash-frozen in liquid nitrogen, and stored at −80 °C for further use. All procedures were approved by the Ethics Committee of the Capital Institute of Pediatrics (DWLL2021004).

### 4.2. Measurement of SBP in the Tail Artery of Rats

According to the user’s manual, SBP and DBP were measured in the tail artery of rats using a BP-2000 intelligent non-invasive sphygmomanometer for SBP. To make the measurement results more accurate, the temperature of the measurement environment was controlled at 37 °C–39 °C. The measurement was performed after the rats were in a stable state, and adaptive measurements were performed 10 times for each rat before the formal measurement experiment. Subsequently, five more formal blood pressure measurements were taken for each rat, and the data were recorded. A difference of <10% between five consecutive readings was considered valid, and the average was recorded for the analysis.

### 4.3. Determination of Serum Hcy Concentrations

Blood samples (10 mL) were collected from the heart of each rat. The blood was stored in tubes containing EDTA anticoagulant, and serum was obtained by centrifugation at 3000 rpm/min for 15 min. The serum was analyzed by a fully automatic biochemical analyzer to determine Hcy concentrations.

### 4.4. Cell Culture and Treatment

EA. hy926 cells (Cell Resource Center, Institute of Basic Medical Sciences, CAMS/PUMC, the manufacturer number is TRX301) were grown in 60 mm Petri dishes in Dulbecco’s Modified Eagle Medium (DMEM, with 4. 5 g/L glucose, L-glutamine, and sodium pyruvate) with 10% FBS. Cells were cultured at 37 °C in 5% CO_2_ and passaged with trypsin-EDTA. Then, the cell culture medium was changed to DMEM with 1% fatty acid-free bovine serum albumin (BSA) and cultured for 24 h before the experiment.

The cells were divided into a control group (Con), methionine treatment groups (Met 0.5 mmol/L and Met 1 mmol/L), and folic acid treatment groups (Met 0.5 mmol/L + folic acid 10 mg/L). The treatment solutions were prepared using serum-free medium, and the cells were treated for 24 h. The control group received an equal volume of sterile PBS. Each group was set up in triplicate.

After treatment, the cells were harvested for total protein and RNA extraction, which were subsequently used for Western blot and quantitative real-time PCR (qRT-PCR) analyses, respectively.

### 4.5. Histopathology

Hematoxylin–eosin staining was carried out on the basis of steps reported previously to assess morphological alterations in the aorta, kidney, and heart. Tissues of each group were fixed with 4% formaldehyde, cut into small pieces with a thickness of 1–2 mm and a length and width of 1 cm, and then rinsed under running water. After dehydration with graded alcohol, the tissue pieces were placed in xylene for transparent treatment and embedded in liquid paraffin. The paraffin-embedded tissue pieces were cut into slices with a thickness of 5 μm using a microtome and dried overnight at 37 °C in an incubator. The dried tissue slices were first dewaxed and then stained with hematoxylin–eosin. The tissue sections were observed by a microscope.

### 4.6. Metabolomics Preparations for Plasma Samples

Plasma samples were obtained from the collected rat blood samples by pre-processing and stored at −80 °C. A volume of 100 μL of the sample was mixed with 400 μL of 50% ice-cold methanol solution. After centrifugation at 12,000 rpm at 4 °C for 10 min, the supernatant was transferred into another 2 mL centrifuge tube and concentrated to dryness by vacuum. The samples were redissolved in 150 µL of 2-chlorophenylalanine (4 ppm) 80% methanol solution, and the supernatant was filtered through a 0.22 µm membrane for liquid chromatography–mass spectrometry. A quality control sample was prepared to assess the analytical variability by mixing equal volumes (20 μL) of the supernatant from each sample. The primary and secondary mass spectra of serum metabolites were matched and identified with the metabolites in the Kyoto Encyclopedia of Genes and Genomes Database and METLIN Metabolite Database. A generalized fold change value (logFC) > 1.5 indicated upregulated enrichment in hypertension, and <0.67 indicated downregulation.

### 4.7. RNA-Sequencing

Total RNA from the rat aorta was extracted with TRIzol reagent (Invitrogen, Carlsbad, CA, USA). Total RNA quality was verified using an Agilent 2100 Bioanalyzer (Agilent Technologies, Waldbronn, Germany). RNA-sequencing libraries were constructed using the NEBNext Ultra RNA Library Prep Kit for Illumina by following the kit instructions. Polymerase chain reaction (PCR) enrichment and purification helped to construct the final libraries, which were evaluated using the Agilent 2100 Bioanalyzer. Raw data were then created using bcl2fastq software (version 2.20), from which raw image files for sequencing were identified and converted to sequencing reads. Clean data were filtered from the raw data. The clean data were aligned with a reference sequence using Hisat2/Tophat. Transcript assembly was performed using Stringtie software (version 2.2.1). The read count and the fragments per kilobase of transcript per million mapped reads of the gene were then calculated.

### 4.8. Protein Extraction and Western Blot Analysis

Cells and tissue samples were lysed using RIPA lysis buffer containing protease inhibitors, and the supernatants were collected by centrifugation. Protein concentrations were measured using a bicinchoninic acid (BCA) protein assay kit (Sangon, Shanghai, China). Equal amounts of protein (30 μg) were separated by SDS-PAGE on 10% polyacrylamide gels and transferred onto polyvinylidene difluoride (PVDF) membranes.

The membranes were blocked with 5% bovine serum albumin (BSA) at room temperature for 1.5–2 h, followed by overnight incubation at 4 °C with primary antibodies, including CYP1A1 (bs-1606R), GPR75 (bs-16263R) (both from Bioss Biotechnology, Beijing, China), and GAPDH (ab8245, Abcam, Cambridge, UK). After washing with TBST, membranes were incubated with HRP-conjugated secondary antibodies for 1 h, including rabbit IgG (H+L)/HRP (ZB-2301) and mouse IgG (H+L)/HRP (ZB-5305), both from ZSGB-Bio (Zhongshan Jinqiao, Beijing, China).

Protein bands were visualized using enhanced chemiluminescence (ECL) reagents and captured with an imaging system. Band intensities were quantified using ImageJ software (version 1.8.0), with GAPDH serving as the loading control for normalization.

### 4.9. RNA Extraction and Quantitative Real-Time PCR (qRT-PCR) Analysis

Total RNA was extracted separately from cells and tissues. For cells, RNA was isolated using Trizol reagent (Invitrogen, Carlsbad, CA, USA), while tissue samples were processed using the RNeasy Mini Kit (74104; Qiagen, Toronto, ON, Canada), according to the manufacturers’ instructions. RNA concentration and purity were assessed using a NanoDrop spectrophotometer (Wilmington, DE, USA).

Equal amounts of total RNA were reverse-transcribed into complementary DNA (cDNA) using the PrimeScript RT reagent kit (Takara, Kyoto, Japan). QRT-PCR was performed in a 10 μL reaction system containing SYBR Green PCR Master Mix (Takara, Japan), diluted cDNA template, gene-specific primers, and DEPC-treated water. Each sample was run in triplicate on an ABI QuantStudio 3 real-time PCR system.

The thermal cycling conditions were as follows: an initial denaturation at 95 °C for 30 s, followed by 40 cycles of 95 °C for 5 s and 60 °C for 30 s. The target genes included *Cyp1a1*, *Gpr75*, *Nras*, *Mek1*, and *Erk1*, with GAPDH serving as the internal control. Relative mRNA expression levels were calculated using the 2^−ΔΔCt^ method.

### 4.10. NHANES

**Figure 7:**
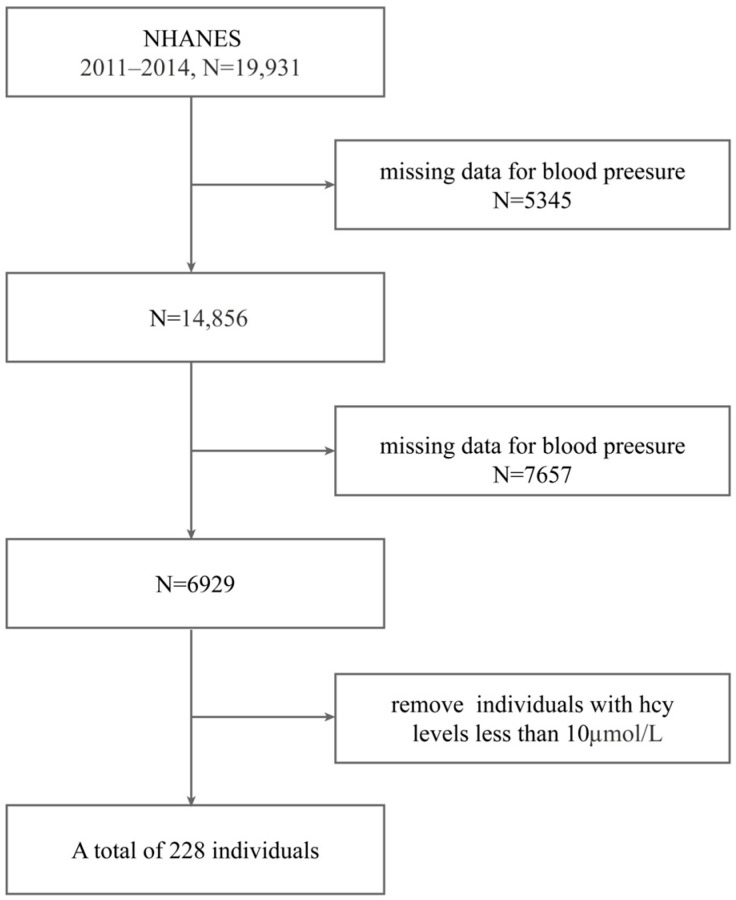
The flow of the participant selection.

The data taken were from the US NHANES database for the hypertensive population from 2011 to 2014. A total of 19,931 data were removed, 5345 blood pressure values were missing, and 7657 population fatty acid data were missing. Based on the definition of H-type hypertension, we classified patients who met the criteria for both hypertension and homocysteine (Hcy) levels greater than 10 µmol/L as having H-type hypertension. A total of 228 individuals were included. We excluded the indicators most directly related to H-type hypertension, namely homocysteine (Hcy) and blood pressure, and used a random forest algorithm to analyze the association between other laboratory indicators and the H-type hypertension population. Please refer to the flowchart for details. See Figure 7 for details.

### 4.11. Statistical Analysis

SPSS 22.0 software was used for the statistical analysis. Measurement data are expressed as the mean and standard deviation.

Blood pressure values of 1–16-week-old rats were compared by analysis of variance. The blood pressure values of 16–20-week-old rats were analyzed by one-way repeated measures analysis of variance, and differences were considered statistically significant at *p* < 0.05. All experiments were repeated at least three times.

The non-targeted metabolomic data were analyzed using Metabo Analyst R. The peak areas were normalized to internal standards. Metabolites with a percentage relative standard deviation of >30% in the quality control samples were excluded, and the remaining data were log-transformed. Important differential metabolites among the groups were defined on the basis of a variable importance in projection value of >1 and a false discovery rate of <0.05. Analysis of variance was used to compare the levels of the metabolites of interest among the groups.

## 5. Conclusions

Our study found that the disorder of plasma AA levels is associated with H-type hypertension. The possible mechanism may be that high hcy levels activate the CYP1A1/20-HETE/GPR75/MAPK axis to elevate blood pressure. This study provides clues for the discovery of new therapeutic targets for H-type hypertension.

## Data Availability

The authors declare that the data supporting the findings of this study are available within this paper. Should any raw data files be needed, they are available from the corresponding author upon reasonable request.

## Supplementary Materials

The following supporting information can be downloaded at https://www.mdpi.com/article/10.3390/ijms26135947/s1.
