# Supplementary material for: CYP1A1/20-HETE/GPR75 Axis-Mediated Arachidonic Acid Metabolism Dysregulation in H-Type Hypertension Pathogenesis
Source: Int J Mol Sci. 2025 Jun 20;26(13):5947. doi: 10.3390/ijms26135947 (PMC12249897; doi:10.3390/ijms26135947)
Supplement: Supplementary file 1 [file ijms-26-05947-s001.zip › ijms-3646324-supplementary.pdf]

A

Weekly Systolic Blood Pressure (SBP) Measurements in Control, HCY, and FA Groups (n = 6 per group)				
**Time**	**Control（n=6）**	**HCY（n=6）**	**FA（n=6）**	**P**
	SBP(mmHg)	SBP(mmHg)	SBP(mmHg)	
**Baseline**	117.13±0.44	117.13±0.44	117.13±0.44	---
Week 6	123.38±2.05	135.00±2.87	132.75±1.82	<0.01
Week 7	122.63±2.20	137.25±2.75	131.25±1.73	<0.001
Week 8	120.75±3.15	136.00±3.09	132.25±2.04	<0.01
Week 9	117.25±3.17	141.25±1.84	141.63±2.25	<0.001
Week 10	116.63±5.28	137.38±0.75	140.00±2.62	<0.001
Week 11	119.38±2.00	143.75±1.55	140.38±2.49	<0.001
Week 12	118.13±2.96	139.88±2.52	141.63±2.58	<0.001
Week 13	111.38±2.20	141.88±1.85	142.13±2.68	<0.001
Week 14	112.50±2.29	144.38±2.33	143.63±2.30	<0.001
Week 15	117.50±3.30	145.50±2.55	147.13±1.98	<0.001
Week 16	118.88±3.20	149.75±1.40	150.25±1.33	<0.001
Week 17			125.00±4.43	<0.001
Week 18			123.13±4.95	<0.001
Week 19			123.13±3.12	<0.001
Week 20			116.00±2.07	<0.001
SBP:mean supine systolic blood pressure				
HCY:high methionine diet				
FA:folic acid				

B

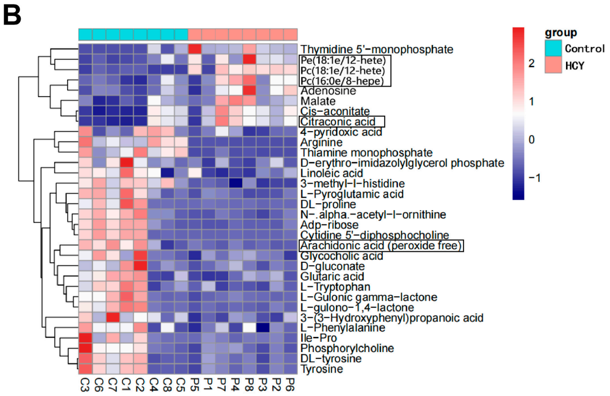

Heatmap of differential metabolites between Control and HCY groups. Each row represents a metabolite, and each column represents a sample (C1–C9: Control group, P1–P9: HCY group). The color scale indicates relative metabolite abundance (z-score), with red representing higher levels and blue representing lower levels. Hierarchical clustering was applied to both metabolites and samples.

Figure S1. (**A**) Weekly Systolic Blood Pressure (SBP) Measurements in Control, HCY, and FA Groups (n = 6 per group). (**B**) Heatmap of Differential Metabolite Profiles in Control and HCY Groups.
